# Ferroptosis induced by environmental pollutants and its health implications

**DOI:** 10.1038/s41420-025-02305-2

**Published:** 2025-01-24

**Authors:** Fu-Han Gong, Liyuan Liu, Xuesheng Wang, Qi Xiang, Xin Yi, Ding-Sheng Jiang

**Affiliations:** 1 Department of Cardiology, Tongren People’s Hospital, Tongren, Guizhou China; 2 Department of Cardiology, Renmin Hospital of Wuhan University, Wuhan, Hubei China; 3 Division of Cardiovascular Surgery, Tongji Hospital, Tongji Medical College, Huazhong University of Science and Technology, Wuhan, Hubei China; 4 Key Laboratory of Organ Transplantation, Ministry of Education; NHC Key Laboratory of Organ Transplantation; Key Laboratory of Organ Transplantation, Chinese Academy of Medical Sciences, Wuhan, Hubei China

**Keywords:** Cell death, Diseases

## Abstract

Environmental pollution represents a significant public health concern, with the potential health risks associated with environmental pollutants receiving considerable attention over an extended period. In recent years, a substantial body of research has been dedicated to this topic. Since the discovery of ferroptosis, an iron-dependent programmed cell death typically characterized by lipid peroxidation, in 2012, there have been significant advances in the study of its role and mechanism in various diseases. A growing number of recent studies have also demonstrated the involvement of ferroptosis in the damage caused to the organism by environmental pollutants, and the molecular mechanisms involved have been partially elucidated. The targeting of ferroptosis has been demonstrated to be an effective means of ameliorating the health damage caused by PM2.5, organic and inorganic pollutants, and ionizing radiation. This review begins by providing a summary of the most recent and important advances in ferroptosis. It then proceeds to offer a critical analysis of the health effects and molecular mechanisms of ferroptosis induced by various environmental pollutants. Furthermore, as is the case with all rapidly evolving research areas, there are numerous unanswered questions and challenges pertaining to environmental pollutant-induced ferroptosis, which we discuss in this review in an attempt to provide some directions and clues for future research in this field.

## Facts

- The major regulatory mechanisms of ferroptosis include the regulation of iron metabolism, the control of reactive oxygen groups by the redox systems, and the metabolism of lipids and amino acids.
- A variety of environmental pollutants (e.g., PM2.5, organic and inorganic pollutants) have been shown to cause damage to the organism through the activation of ferroptosis. Furthermore, activation of ferroptosis by ionizing radiation, particularly radiotherapy, has been employed as a therapeutic approach to target tumors.
- Interventions that target ferroptosis by restoring iron homeostasis, enhancing antioxidant defenses, and preventing lipid peroxidation have the potential to serve as effective strategies to mitigate the adverse health effects of environmental pollutants and improve outcomes for affected populations.

## Open questions

- Which environmental pollutants cause damage to organisms by activating ferroptosis, and how can this damage be prevented by targeting ferroptosis?
- Do distinct molecular mechanisms exist by which different environmental pollutants activate ferroptosis?
- How might the regulation of ferroptosis in cancer patients undergoing radiotherapy help reduce the harmful side effects of treatment while preserving its tumor-killing efficacy?
- The issue of how to address the gap whereby organisms are often exposed to multiple pollutants at the same time, while the majority of current research focuses on a single pollutant, is one that has yet to be fully explored.

## Introduction

Exposure to environmental pollutants is one of the most serious problems that affect human health. Pollution caused by the overexploitation of natural resources and the excessive release of harmful chemicals at a rate that exceeds nature’s ability to replenish itself ultimately leads to the contamination of the physical and biological components of the planet [1]. The global burden of water, air, metal, and chemical pollution has increased in conjunction with the modern expansion of industrialization, fossil fuel combustion, and mechanized agriculture [2]. The number of deaths attributable to pollution continues to increase as populations around the world are exposed to rising levels of environmental pollutants [2]. In 2016, the World Health Organization (WHO) estimated that 13.7 million deaths per year were attributable to modifiable environmental risks, representing 24% of the global total. It has been documented that pollution represents the most significant environmental risk factor for premature mortality globally, and the second leading cause of noncommunicable diseases worldwide after smoking. This contributes to an escalating “pandemic” of cardiovascular disease, cancer, and respiratory disease [2, 3]. It can be reasonably deduced, therefore, that nearly a quarter of the global burden of disease could be prevented by the creation of healthier environments.

A substantial body of research has demonstrated that prolonged exposure to fine particulate matter (PM2.5), the most significant environmental health risk factor, is strongly associated with the advancement of diseases affecting the cardiovascular system, respiratory system, nervous system, digestive system, and reproductive system. The heart and lungs are the most commonly affected organs, with a range of adverse effects including heart and lung dysplasia, irregular heartbeat, worsened asthma, reduced lung function, and exacerbated respiratory symptoms [4]. It is frequently observed that environmental exposures are associated with the etiology of disease, whereby they interact with or induce DNA mutations and epigenetic alterations. The intricate, non-linear interactions between environmental pollutants and the genome, epigenome, transcriptome, epitranscriptome, proteome, and metabolome result in adverse health outcomes [2]. In recent years, there has been a growing recognition of the impact of environmental pollutants on human health through the induction of programmed cell death, which can result in tissue or organ injury. For example, air pollutants, particularly PM2.5, have been demonstrated to induce a number of different types of programmed cell death, including apoptosis, pyroptosis, necrosis, and ferroptosis. These processes ultimately contribute to the development of disease [5, 6]. Among these, ferroptosis, a mode of programmed cell death discovered in the last decade, is being increasingly investigated as a potential mechanism by which environmental pollutants affect human health [7, 8]. In addition to air pollutants, numerous other pollutants, including microplastics, bisphenol A, heavy metals, and radiation, have been demonstrated to influence human health by regulating ferroptosis [7]. However, a comprehensive review synthesizing the findings of these studies is currently lacking. Consequently, this review aims to provide a summary and analysis of the impact of pollutants on human health through ferroptosis, along with an examination of the underlying mechanisms.

## Key mechanisms in ferroptosis regulation

**Fig. 1:**
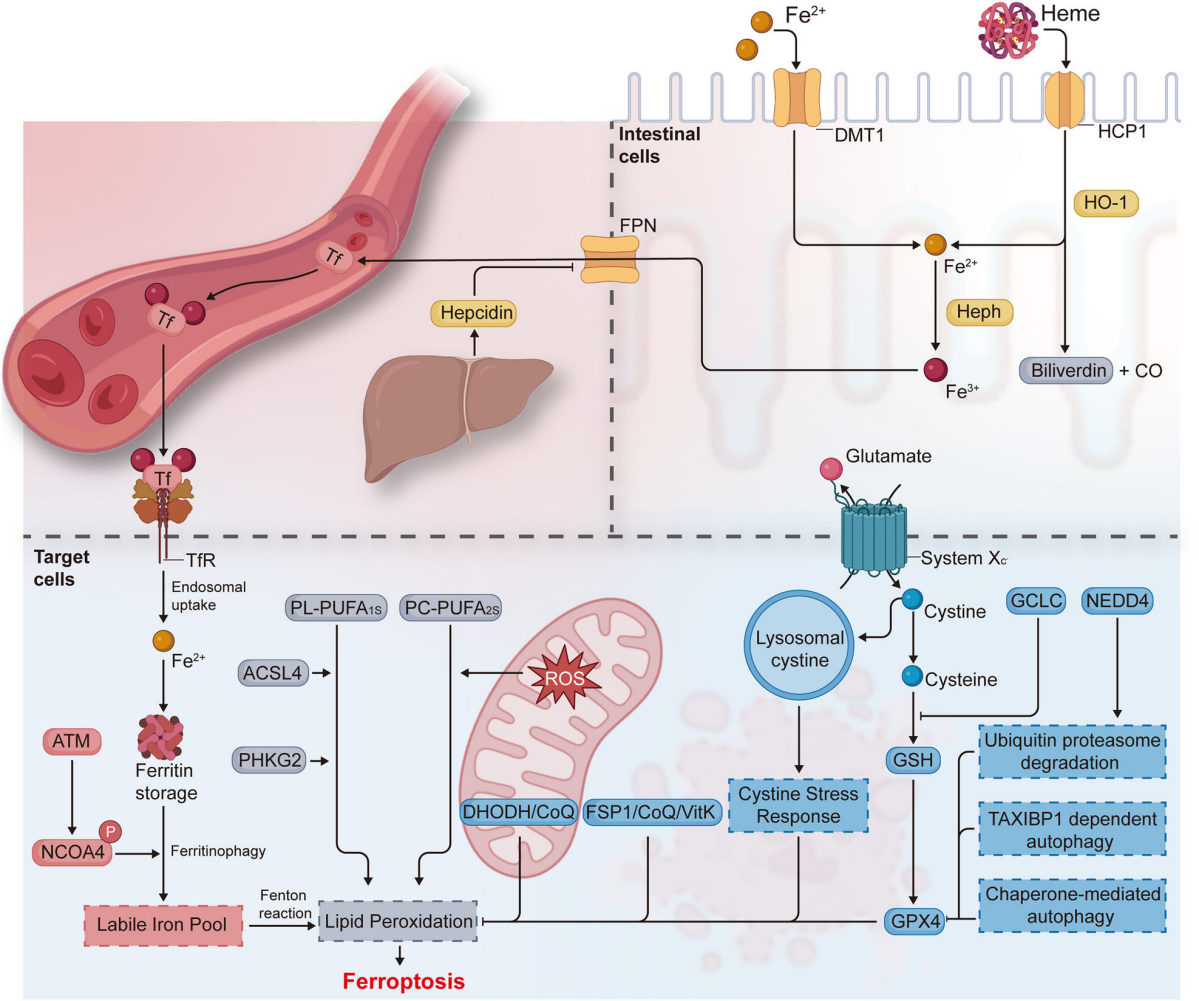
The mechanisms of ferroptosis. Dietary sources of iron, as well as heme iron, are absorbed through the small intestine and transported into the circulation where they bind to transferrin (Tf) and are transported to the organs of the body. Iron is mainly stored in ferritin after entering target cells, which can be released when needed through nuclear receptor coactivator 4 (NCOA4)-regulated ferritinophagy. The Fenton reaction of divalent iron ions with polyunsaturated fatty acids (PUFAs) leads to lipid peroxidation, which in turn induces ferroptosis. There are also many important defense systems that prevent cells from undergoing ferroptosis, including System Xc^−^ (xCT)- glutathione peroxidase 4 (GPX4)-glutathione (GSH), ferroptosis suppressor protein 1 (FSP1)-CoQ, FSP1-VitK, dihydroorotate dehydrogenase (DHODH)-CoQ, which independently or synergistically inhibits the onset of ferroptosis. This figure was created using BioRender (https://biorender.com/).

Ferroptosis is an iron-dependent form of programmed cell death, typically characterized by lipid peroxidation. It is not only involved in the pathological processes of various diseases including cardiovascular diseases, tumors, neurodegenerative diseases, and ischemia/reperfusion injury but also plays a physiological function in regulating the vulnerability of human hematopoietic stem cells [9–17]. The current research suggests that ferroptosis is regulated by three main mechanisms: (1) the regulation of iron absorption, storage, and transport, (2) the control of reactive oxygen species (ROS) by the redox system, and (3) the mechanisms of lipid (e.g., phospholipids, fatty acids, and cholesterol) and amino acid (e.g., cysteine) metabolism [18] (Fig. 1).

### Iron absorption, storage, and transport

The intracellular formation of hydroxyl radicals, a process known as the Fenton reaction (Fe^2+^ + H_2_O_2_ → Fe^3+^ + OH^−^ + OH^⋅^), is initiated by the reaction of ferrous ions with peroxides [18]. In the absence of scavenging, the resulting hydroxyl radicals have the potential to cause lipid peroxidation, damage the plasma membrane, and ultimately lead to ferroptosis. The primary source of iron is dietary, and free divalent or heme iron in the intestine is taken up by intestinal cells via divergent metal transporter 1 (DMT-1) and heme carrier protein 1 (HCP-1), respectively [19, 20]. Ferrous ions are oxidized by hephaestin (Heph) and released into the capillaries via ferroportin (FPN, also known as SLC40A1), which is located on the basolateral membrane of the intestinal epithelium [20]. Hepcidin, a hormone primarily synthesized and secreted by hepatocytes, binds to and degrades FPN, leading to intracellular iron retention and a decrease in circulating iron levels [21, 22]. Redox-inactive Fe^3+^ in the circulation was bound by the iron carrier, transferrin (Tf), and subsequently taken up by Tf receptor (TfR)-mediated endocytosis, delivering iron to tissues and organs [23]. Furthermore, TfR is regarded as a specific marker for ferroptosis [24]. Normally, a labile iron pool (LIP) is present within the cell, comprising a reservoir of chelatable and redox-active iron that serves to minimize the potential for ROS formation [25, 26]. Ferritin, comprising 24 subunits of two types, ferritin heavy chain (FTH) and ferritin light chain (FTL), is aprincipal iron storage protein within the cytoplasm [27]. The degradation of ferritin-bound iron and its subsequent release for enzymatic reactions is facilitated by a process known as NCOA4 (nuclear receptor coactivator 4)-mediated ferritinophagy [28, 29]. Additionally, the Ser/Thr protein kinase ATM (ataxia telangiectasia mutated) regulates intracellular labile-free iron by phosphorylating NCOA4, which facilitates the interaction between NCOA4 and ferritin, thus maintaining ferritinophagy [30]. The iron exporter FPN is also a crucial regulator of iron homeostasis [31]. Iron overload induces ferroptosis.

### Redox systems

The Fenton reaction is responsible for the production of copious quantities of hydroxyl radicals in cases of iron overload. A variety of defense systems are in place within organisms to counteract excessive ROS before they cause damage to the body. Glutathione peroxidase 4 (GPX4) is a selenoenzyme that utilizes glutathione (GSH) as a cofactor to prevent lipid peroxidation and ferroptosis during periods of elevated oxidative stress [32, 33]. GPX4 overexpression resulted in the inhibition of the lethality of many ferroptosis inducers, whereas GPX4 knockdown promoted the lethality of these inducers but not of compounds with other lethal mechanisms in cells. This indicated that GPX4 is an essential regulator of ferroptosis [33]. Furthermore, the prevention of hydroperoxide-induced ferroptosis requires the utilization of selenium by GPX4 [34]. It is therefore evident that the regulation of GPX4 expression and stability is of critical importance in the context of ferroptosis. It is anticipated that the precise regulation of GPX4 will facilitate the targeting of ferroptosis in the treatment of diseases. Erastin has been observed to upregulate the expression of lysosome-associated membrane protein 2a (LAMP2a), which has the effect of enhancing the degradation of GPX4 via the chaperone-mediated autophagy pathway, thereby triggering ferroptosis [35]. Similarly, exogenous copper has been observed to increase GPX4 ubiquitination and facilitate TAX1BP1 (Tax1 binding protein 1)-dependent GPX4 autophagic degradation, thereby enhancing ferroptosis in pancreatic cancer tumors [36]. Furthermore, the ubiquitin-proteasome degradation pathway plays a pivotal role in regulating GPX4 stability. For instance, dopamine quinone (DAQ) and α-synuclein facilitate neural precursor cell expressed developmentally down-regulated protein 4 (NEDD4)-mediated GPX4 ubiquitin-proteasome degradation, thereby promoting ferroptosis in neurons during Parkinson’s disease (PD) [37]. DMOCPTL, a derivative of natural product parthenolide, has also been shown to induce GPX4 ubiquitination and reduce GPX4 protein levels, thereby promoting ferroptosis in triple-negative breast cancer cells [38]. Cystine is transported into cells via the cystine-glutamate antiporter system Xc^-^ (SLC7A11/SLC3A2 heterodimer), which serves as a rate-limiting substrate for the synthesis of GSH [39]. Thus, it is expected that cystine starvation can induce ferroptosis [11–13]. Badgley and colleagues showed that oxidized cysteine (cystine) import is a crucial factor in the development of pancreatic ductal adenocarcinoma (PDAC). Furthermore, they showed that SLC7A11 deletion inhibits PDAC growth by inducing tumor ferroptosis in mice [40]. A recent study has indicated that lysosomal cystine may modulate the susceptibility of tumors to ferroptosis through the cysteine stress response [41]. Lysosomal cystine levels were regulated by the major facilitator superfamily domain containing 12 (MFSD12)-mediated import and the lysosomal cystine transporter (CTNS)-mediated efflux and were sensed by the AhR-kynurenine pathway [41–43]. It is noteworthy that cystine starvation has been observed to induce γ-glutamyl-peptide accumulation via the NFE2-like BZIP transcription factor 2 (NRF2)-glutamate-cysteine ligase catalytic subunit (GCLC) axis, thereby preventing ferroptosis in a GSH-independent manner in non-small cell lung cancer (NSCLC) cells [44]. In light of these findings, it can be concluded that the cystine-system Xc^-^-GSH-GPX4 axis plays a pivotal role in antioxidant defense and the regulation of ferroptosis.

Furthermore, a number of additional pathways have been identified that regulate ferroptosis in the organism, operating in parallel with the canonical glutathione-based GPX4 pathway [14]. The ferroptosis suppressor protein 1 (FSP1, also known as AIFM2)-coenzyme Q_10_ (CoQ) pathway was recently identified by two independent research teams as a means of conferring protection against GPX4-deficiency-induced ferroptosis [45, 46]. Furthermore, the plasma membrane localization of FSP1 is crucial for its antioxidant effects, whereby it functions as an oxidoreductase that reduces CoQ, thereby preventing the propagation of lipid peroxides [45]. It has been demonstrated that the ubiquitination of residues K322 and K366 via the K63 linkage of FSP1 and myristoylation of FSP1 facilitate the translocation of FSP1 to the plasma membrane [45, 47]. In addition to CoQ, vitamin K is a substrate for FSP1, which reduces vitamin K to hydroquinone, thereby inhibiting lipid peroxidation and ferroptosis [48]. It is noteworthy that Nakamura et al. discovered that 3-phenylquinazolinones are potent FSP1 inhibitors, inducing the subcellular relocalization of FSP1 from the membrane but not inhibiting FSP1 enzyme activity to accelerate ferroptosis via a phase separation mechanism [49]. Furthermore, the myristoylated N-terminus, distinct amino acid residues, and intrinsically disordered, low-complexity regions of FSP1 were identified as essential for FSP1-dependent phase separation [49]. Dihydroorotate dehydrogenase (DHODH), which is located at the inner mitochondrial membranes, reduces CoQ to ubiquinol, thereby scavenging phospholipid radicals and preventing ferroptosis in tumor cells, Conversely, its inhibitor brequinar has been demonstrated to suppress the growth of GPX4^low^ tumor cells through the induction of ferroptosis [50]. Although the hypothesis that DHODH-CoQ acts independently of cytosolic GPX4 and FSP1 to inhibit ferroptosis was initially proposed, Mishima et al. argue that this conclusion remains controversial. They found that the inhibitors of DHODH had a significant ferroptosis-promoting effect only at high concentrations that also inhibited FSP1 [51]. Consequently, further studies are required to elucidate the mechanisms that regulate ROS production and the regulatory networks between redox systems.

### Lipid and amino acid metabolism

The process of lipid metabolism plays a pivotal role in determining the susceptibility and execution of ferroptosis, particularly with regard to phospholipid peroxidation. It is hypothesized that phospholipids with a single polyunsaturated fatty acyl tail (PL-PUFA1s) are the primary drivers of ferroptosis [52]. A recent study indicates that diacyl-PUFA phosphatidylcholines (PC-PUFA_2_s) are also essential for ferroptosis, as they interact with the mitochondrial electron transport chain to generate ROS, which initiates lipid peroxidation [52]. Yang et al. demonstrated that phosphorylase kinase G2 (PHKG2) facilitates bis-allylic polyunsaturated fatty acid (PUFA) peroxidation by regulating iron availability to lipoxygenases, thereby enhancing ferroptosis [53]. The elongation of very long-chain fatty acid protein 5 (ELOVL5) and fatty acid desaturase 1 (FADS1)-regulated biosynthesis of arachidonic acid (AA) and adrenic acid (AdA), which are substrates for lipid peroxidation, represents a critical checkpoint in ferroptosis. Furthermore, AA supplementation has been demonstrated to increase sensitivity to ferroptosis [54]. The ACSL4 (acyl-CoA synthetase long-chain family member 4) was identified as an essential component for the execution of ferroptosis by two independent screening approaches: microarray analysis of ferroptosis-resistant cell lines and a genome-scale CRISPR-guided genetic screen. ACSL4 is involved in shaping the cellular lipid composition, in particular the enrichment of long polyunsaturated ω6 fatty acids in cell membranes [55]. An unbiased multi-omic analysis identified cyclin-dependent kinase inhibitor 2 A (CDKN2A) as a regulator of lipid metabolism. CDKN2A enhances oxidizable PUFA sequestration into lipid droplets, thereby protecting against ferroptosis [56]. It is unclear which cellular membranes are the primary sites of lipid peroxidation during ferroptosis, given that PUFA moieties could be incorporated into membranes throughout the cell. The most recent research by Stockwell and his team posits that lipid peroxidation occurs initially in the endoplasmic reticulum (ER) membrane and subsequently in the plasma membrane [57]. Furthermore, the maintenance of cholesterol homeostasis and peroxisome-driven ether-linked phospholipid biosynthesis are essential for the occurrence of ferroptosis [58, 59]. Recent studies have shown that enzymes (e.g., DHCR7, MSMO1, CYP51A1, EBP, and SC5D) involved in distal cholesterol biosynthesis have been identified as having a critical role in the regulation of ferroptosis, with the ability to dictate the level of 7-dehydrocholesterol (7-DHC) [60, 61]. The accumulation of 7-DHC protects plasma and mitochondrial membranes from phospholipid autoxidation and subsequent fragmentation, thereby preventing ferroptosis and promoting tumor growth [60, 61].

Furthermore, intercellular interactions and energy stress-mediated AMPK activation are essential for the regulation of ferroptosis [62, 63]. While studies on the regulatory mechanisms of ferroptosis are advancing at a rapid pace and many crucial mechanisms are being elucidated, further investigation into the pathological factors that induce or inhibit ferroptosis and the potential of targeting ferroptosis for the prevention of related diseases is still required.

### Ferroptosis caused by environmental pollutants and its health implications

**Fig. 2:**
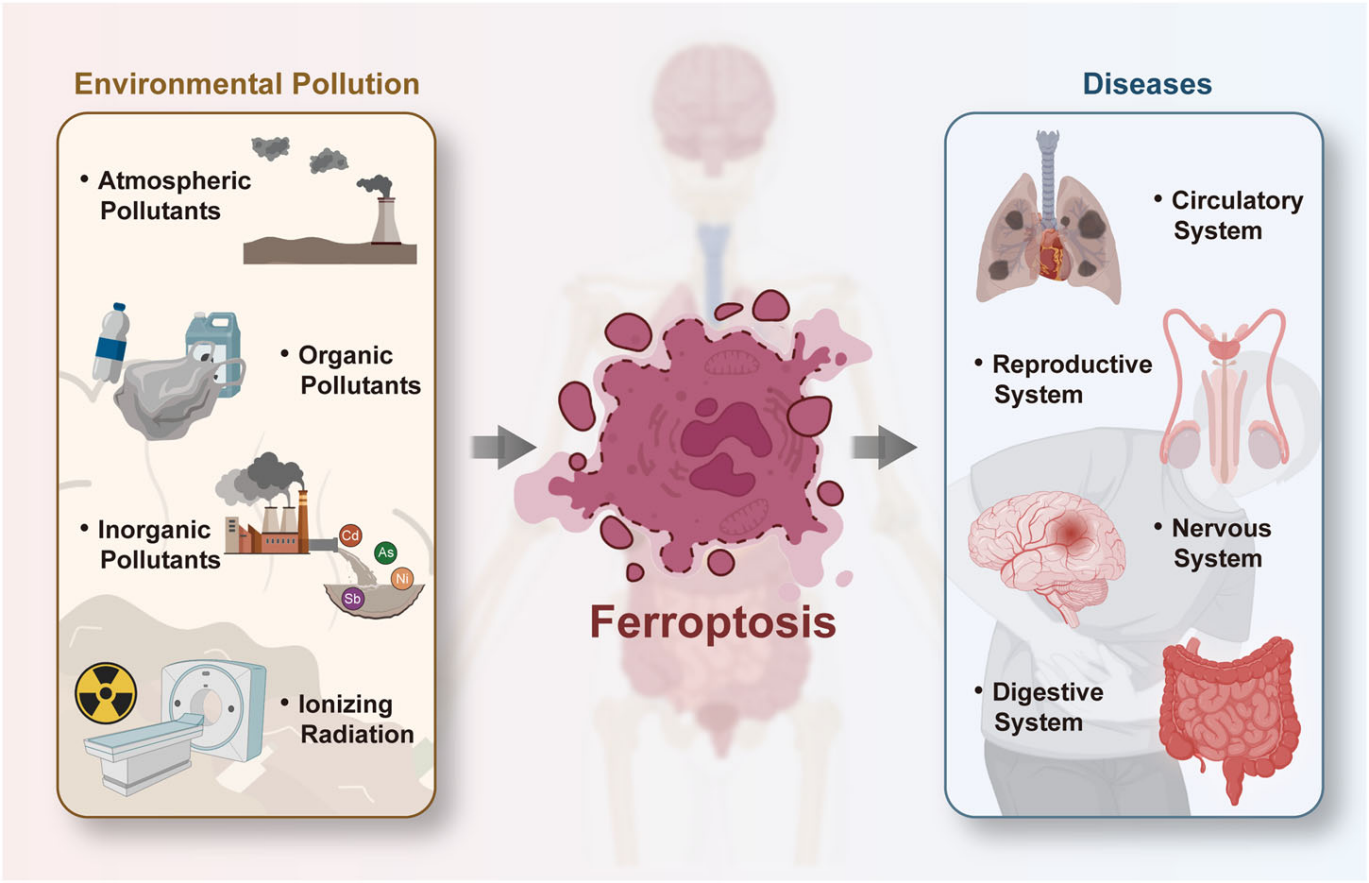
Adverse health effects of environmental contaminants by inducing ferroptosis. Ferroptosis can be induced by various environmental pollutants (e.g., atmospheric pollutants, organic pollutants, inorganic pollutants, ionizing radiation) to affect the health of the circulatory, reproductive, nervous, and digestive systems. This figure was created using BioRender (https://biorender.com/).

A number of studies conducted over the past few years have confirmed that environmental pollutants cause cellular ferroptosis, which has implications for human health. Of these, the effects of atmospheric pollutants, organic pollutants, inorganic pollutants, and radiation on ferroptosis have been the subject of the most extensive research (Fig. 2). This review presents a summary and discussion of the progress made in this field of research.

### Ferroptosis induced by atmospheric pollutants and its health implications

#### Circulatory system

The effects of atmospheric pollutants on human health are long-lasting and detrimental. These pollutants infiltrate the respiratory tract, causing a range of respiratory illnesses [64]. Indeed, PM2.5 has been demonstrated to inflict damage upon a multitude of bodily systems through the induction of oxidative stress, inflammation, and regulated cell death (including apoptosis, necroptosis, and pyroptosis) [65, 66]. In recent years, an increasing number of studies have shown that PM2.5 affects respiratory health by inducing ferroptosis, which can result in conditions such as lung injury, lung toxicity, and pulmonary fibrosis [67–69]. Firstly, PM2.5 has been demonstrated to promote the ferroptosis and senescence of type II alveolar epithelial (AT2) cells, which represent the primary line of defense against a range of air pollutant particles. This process is facilitated by proteasomal degradation of SIRT3, which enhances the acetylation of P53 (P53^K320ac^) and its transcriptional activity [70]. In a PM2.5-induced acute lung injury (ALI) mouse model, iron accumulation, increased lipid peroxidation, and decreased SLC7A11 expression were detected, which were partially reversed by ferroptosis inhibitors [67]. PM2.5 activated the AMP-activated protein kinase (AMPK)-BECN1 signaling pathway, and BECN1 directly interacted with SLC7A11 to promote ferroptosis. Moreover, the downregulation of BECN1 or the inhibition of AMPK was found to attenuate PM2.5-induced ferroptosis and ALI [67]. Furthermore, tectoridin, astragaloside IV, melatonin, rosavin, and sipeimine were identified as having the capacity to ameliorate PM2.5-induced lung injury by inhibiting ferroptosis in a manner that is dependent on Nrf2 [66, 71–74]. Furthermore, astaxanthin has been demonstrated to protect against PM2.5-induced lung injury by inhibiting both ferroptosis and apoptosis [75]. In addition to lung injury, PM2.5 has been reported to exacerbate the fibrotic process in pre-existing pulmonary fibrosis and to exacerbate asthma by activating ferroptosis [69, 76].

PM2.5 has been demonstrated to pose a threat to cardiovascular health in a number of ways, including the stimulation of an inflammatory response and oxidative stress. Furthermore, there is a growing body of evidence associating PM2.5 exposure with an increased risk of developing a number of cardiovascular diseases, including hypertension, arrhythmias, ischemic heart disease, heart failure, and atherosclerosis [77, 78]. Recently, there have been reports indicating that ferroptosis may also play a role in mediating cardiovascular injury induced by PM2.5. For example, Ren et al. reported that pretreatment with sesquiterpene inhibited ferroptosis and thus attenuated PM2.5-induced cardiac fibrosis by increasing antioxidant capacity and reducing inflammation [79]. Furthermore, it was demonstrated that astaxanthin exerts a protective effect against PM2.5-induced cardiomyocyte injury by inhibiting ferroptosis [78]. In addition, transcription factor Yin Yang 1 (YY1) has been demonstrated to influence the development of myocardial fibrosis in response to PM2.5 exposure, through its regulation of ferritinophagy [80]. It is well known that the onset of ferroptosis requires elevated levels of ferrous ions, which induce lipid peroxidation and ultimately result in cell death. While these studies indicate that ferroptosis may play a role in PM2.5-induced myocardial injury, several questions remain to be addressed. Firstly, it would be beneficial to ascertain whether there is an increase in ferrous ion levels during this process. Secondly, it would be helpful to determine in which cell types lipid peroxidation and cell death occur. Finally, although ferroptosis inhibitors have been shown to reverse PM2.5-induced myocardial injury, it would be interesting to investigate whether this function is dependent on ferroptosis or if it is simply the scavenging of peroxides.

#### Reproductive system

It is evident that exposure to PM2.5 is a significant risk factor for the development of reproductive disorders and congenital anomalies [81, 82]. PM2.5 is capable of crossing a number of biological barriers, including the blood testis barrier, the epithelial barrier, the placental barrier, and others. Once inside reproductive tissues, it accumulates and induces a number of adverse effects, including oxidative stress, inflammation, apoptosis, and barrier dysfunction. These effects collectively contribute to reproductive toxicity. On the other hand, PM2.5 has also been demonstrated to impact fertility by disrupting hormonal balance [83]. A reduction in sperm quality has been observed as a consequence of PM2.5-induced reproductive toxicity [83, 84]. PM2.5-induced redox imbalance, DNA damage response, ferroptosis, and cell cycle arrest have been demonstrated to reduce the number of Sertoli cells (which are involved in the spermatogenesis process) in the seminiferous tubules in mice. This is achieved by activating xenobiotic metabolism and reducing the GSH/GSSG ratio [82]. Moreover, PM2.5 treatment was observed to induce a dose-dependent elevation in cellular ROS, iron overload, ferritin accumulation, and lipid peroxidation in testicular Leydig cells. This resulted in the ferroptosis of Leydig cells, testicular dysfunction, and a reduction in sperm quality. However, these effects were largely reversed by ferrostatin-1 in mice [85]. Similarly, a reduction in GPX4 expression, an increase in ACSL4 and ALOXE3 expression, an accumulation of iron and lipid peroxidation were observed in spermatocytes following PM2.5 treatment. The administration of the iron chelator deferoxamine mesylate (DFOM) and the ferrostatin-1 significantly attenuated the PM2.5-induced damage to the male reproductive system [86]. Furthermore, a reduction in sperm count and fertility, along with redox dysregulation, mitochondrial dysfunction, impaired cellular iron metabolism, and a significant increase in ferroptosis during sexual maturation, were observed in juvenile male rats exposed to PM2.5 [87]. It has been demonstrated that exposure to 1-nitropyrene (1-NP), which is widely recognized as a key toxic component of PM2.5, induces placental trophoblast ferroptosis via the activation of the CYP1B1/ERK pathway. This contributes to fetal growth restriction in pregnant mice [88]. In light of these findings, it can be posited that ferroptosis plays a role in mediating the damage caused to the reproductive system by PM2.5.

#### Nervous system

Previous studies have shown that exposure to PM2.5 is associated with adverse neurological effects, including the development of epilepsy, Parkinson’s disease, and cognitive impairment [89]. Recent studies have indicated that the effects of PM2.5 on the nervous system may be mediated by ferroptosis of neuronal cells. The increased cell death observed in PM2.5-treated Neuro-2a (N2A) and SH-SY5Y neurons was accompanied by a decrease in GPX4 and ferritin heavy chain expression and an increase in transferrin receptor protein (TFRC) expression. However, these effects were reversed by the ferroptosis inhibitor ferrostatin-1 [90]. Similarly, Guo et al. demonstrated that PM2.5 treatment induced ferroptosis, as evidenced by increased iron, MDA, and lipid ROS levels; decreased GSH levels and activity of GSH-PX and SOD; and decreased HO-1, NRF2, SLC7A11, and GPX4 expression levels in SH-SY5Y cells [91]. More interestingly, Mei et al. demonstrated that PM2.5 induced hippocampal neuronal ferroptosis by increasing intracellular ferrous ions and lipid peroxidation levels in an NRF2-dependent manner, thereby exacerbating seizure symptoms and cognitive dysfunction [92]. However, Wei et al. demonstrated that PM2.5 exposure disrupted autophagic flux and thus induced cell death by impairing lysosomal function. Their results showed that impairment of autophagic flux upregulates the expression of antioxidant genes by activating the NRF2-P62 pathway and inhibits the degradation of GPX4 and ferritin, thereby reducing susceptibility to ferroptosis [93]. Therefore, although the evidence is still inconclusive, it seems reasonable to conclude that ferroptosis plays a significant role in PM2.5-induced neurological impairment and neurological disorders.

#### Other diseases

Furthermore, exposure to PM2.5 has been demonstrated to exert significant effects on a range of other diseases. For example, exposure to PM2.5 has been demonstrated to result in the accumulation of labile iron, an increase in ROS and lipid peroxidation, and the induction of ferroptosis in human nasal epithelial cells. This process is mediated by the activation of AMPK-mediated autophagy. The reversal of the effects of PM2.5 on ferroptosis by the inhibition of AMPK through the use of Compound C or siRNA provides further evidence of the involvement of this pathway in the observed effects [94]. Furthermore, ferroptosis plays a role in PM2.5-induced damage to the inner blood-retinal barrier, as evidenced by iron overload and excessive lipid oxidation, as well as increased expression levels of PTGS2 and FTH1 [95]. Furthermore, PM2.5 has been demonstrated to induce ferroptosis in human small intestinal cells, which is implicated in the pathogenesis of inflammatory bowel disease [96].

**Fig. 3:**
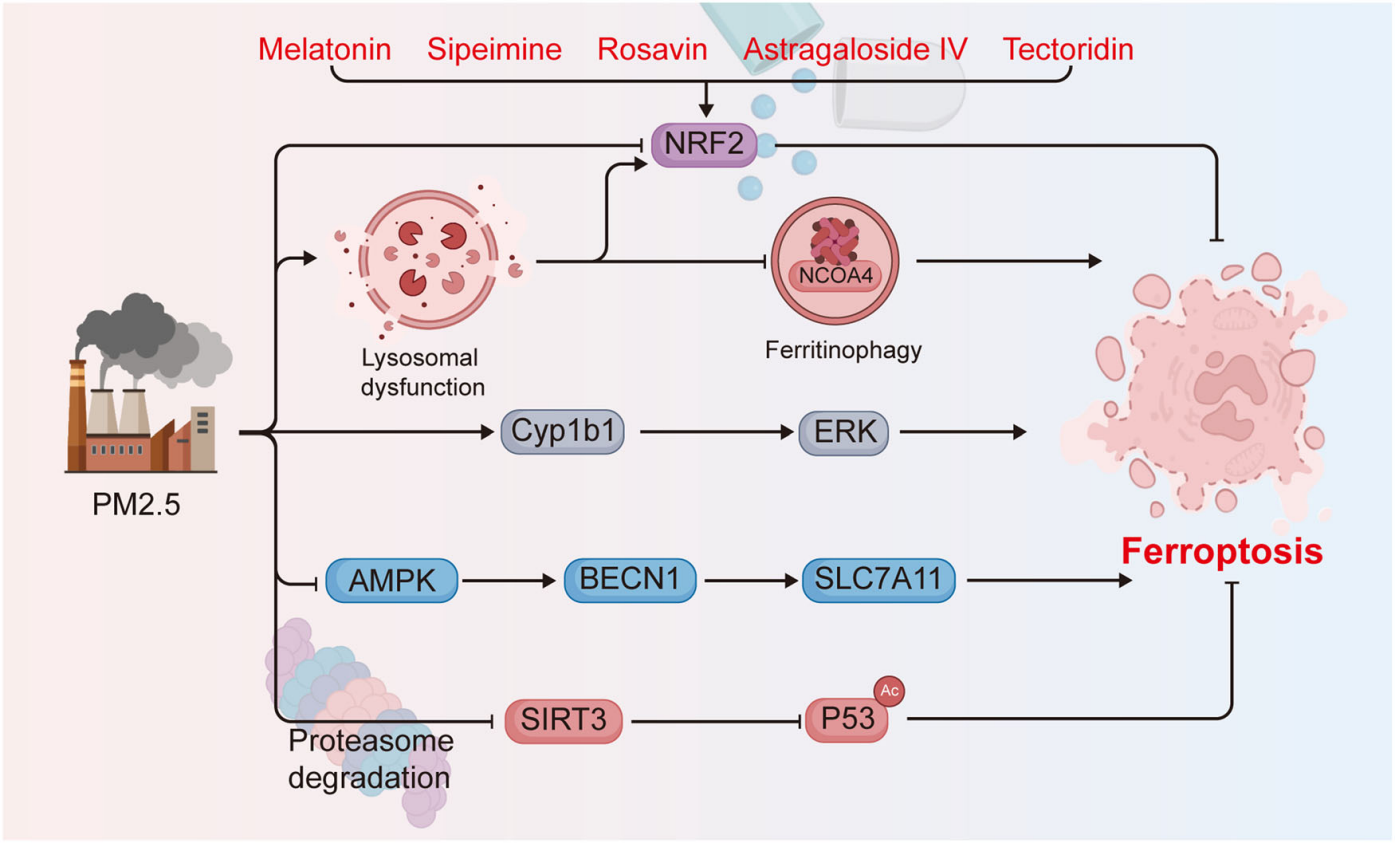
The molecular mechanisms of PM2.5-regulated ferroptosis. PM2.5 exposure promotes ferroptosis by regulating ferritinophagy, NRF2, CYP1B1, AMPK, and SIRT3, whereas tectoridin, astragaloside IV, melatonin, rosavin, and sipeimine can prevent PM2.5-induced ferroptosis in an NRF2-dependent manner. This figure was created using BioRender (https://biorender.com/).

In conclusion, ferroptosis represents a pivotal mechanism underlying PM2.5-induced injury and disease. Targeted inhibition of ferroptosis may offer a novel strategy for combating PM2.5-associated diseases (Fig. 3).

### Ferroptosis induced by organic pollutants and its health implications

#### Microplastics and nanoplastics

Microplastics (MPs) and nanoplastics (NPs) are pervasive contaminants of living environments, including the atmosphere, soil, water, and oceans. These contaminants pose a significant and underestimated threat to human health [97]. Exposure to MPs has been demonstrated to result in hepatic metabolic dysfunction and the enhancement of glutamine and glutamate synthesis. This, in turn, has been shown to impair the Nrf2-Keap1-HO-1/NQO1 pathway, leading to the induction of autophagy-dependent ferroptosis and apoptosis of Purkinje cells. This process ultimately causes neurological dysfunction in chickens [97]. In microglia (BV2) cells, NPs have been observed to activate the JNK/HO-1 pathway, which in turn induces ferrous ion accumulation, lipid peroxidation, and ferroptosis [98]. In addition to neurotoxicity, MPs/NPs have been demonstrated to exhibit developmental toxicity. For example, exposure to polystyrene nanoparticles (PS-NPs) during pregnancy has been demonstrated to induce ferroptosis, which in turn leads to histological changes in the small intestine of offspring of both sexes. This process is mediated by the upregulation of ROS and the downregulation of the expression of GPX4, FTH1, and FTL [99]. Of particular interest is the observation that PS-NP-induced ferroptosis has a greater impact on the development of female offspring than on male offspring. NP exposure has been demonstrated to result in a reduction in the birth weight of female offspring, indicating a heightened sensitivity to NP-induced toxicity in this sex [99]. The administration of NPs has been shown to cause severe developmental toxicity, as evidenced by spinal deformity and organ edema, through the activation of ferroptosis, apoptosis, and inflammation in zebrafish larvae. However, these effects can be largely reversed by the administration of sodium nitroprusside (SNP) solution [100]. In contrast, Li et al. reported that carboxyl-modified polystyrene nanoparticles (CPS) enter cells and fuse with lysosomes in a macropinocytosis-dependent manner to induce lysosomal stress. This leads to the translocation of transcription factor EB (TFEB) into the nucleus, where it promotes the expression of SOD, thereby reducing ROS and lipid peroxidation and inhibiting ferroptosis [101].

#### Bisphenol A (BPA)

Bisphenol A (BPA) is an environmental endocrine disruptor that is widely used in food/beverage containers and polycarbonate plastic manufacturing [102]. An increasing body of evidence indicates a correlation between BPA exposure and adverse human health outcomes, including insulin resistance, obesity, cardiac malformations, and cardiac structural changes [103]. Recent studies indicate that ferroptosis may be a mediator of the adverse health effects of BPA in humans. For example, exposure to BPA has been demonstrated to disrupt hepatic lipid metabolism, including the elevation of triglyceride, total cholesterol, and low-density lipoprotein cholesterol levels, the reduction of high-density lipoprotein cholesterol levels, and the alteration of fatty acid metabolism. Conversely, BPA treatment has been observed to elevate intracellular iron levels and promote lipid peroxidation, which ultimately culminates in hepatic ferroptosis. It is also noteworthy that the effects of BPA on hepatic lipid metabolism and ferroptosis are achieved through the activation of G-protein coupled estrogen receptor (GPER) expression [104]. The administration of both low-dose (40 mg/(kg⋅bw)) and high-dose (120 mg/(kg⋅bw)) BPA has been demonstrated to downregulate the expression of SLC7A11 and SLC3A2, thereby inducing ferroptosis and resulting in the abnormal development of the fetal heart in mice [103]. In addition to the liver and heart, BPA has also been demonstrated to damage the kidney. BPA induces ferroptosis of renal tubular epithelial cells in the kidney by promoting ferritinophagy through the activation of the AMPK-mTOR-ULK1 pathway. This damage can be reversed by the ferroptosis inhibitors ferrostatin-1 and desferrioxamine, and the autophagy inhibitor chloroquine [105]. A joint bioinformatics analysis of data from the Comparative Toxicogenomics Databases (CTD), NCBI-Gene Expression Omnibus (GEO), and TCGA databases revealed that alternative bisphenols (BPS, BPF, and BPAF) are the factors contributing to prostate cancer progression and that ferroptosis is involved in this process [106].

#### Phthalates

Phthalates, exemplified by di(2-ethylhexyl) phthalate (DEHP) and dibutyl phthalate (DBP), constitute a class of plasticizers and solvents that are pervasively employed in food packaging, children’s toys, and medical instruments. This is done with the objective of enhancing the malleability and flexibility of the material [107]. Given their persistent and toxic endocrine-disrupting properties and their prevalence in everyday products, phthalates have become a significant public health concern [108]. Following a 28-day period of DEHP treatment, mice displayed indications of cardiac injury, including the loss of mitochondrial cristae, an increase in lipid peroxidation and ferrous ion levels, and a subsequent rupture of cardiomyocytes. These effects were primarily attributed to DEHP-induced activation of the Nrf2/HO-1 pathway, which resulted in the induction of ferroptosis in cardiomyocytes [109]. Additionally, DEHP was observed to induce liver injury through the activation of ferroptosis, which was reversed by apigenin (APG) through the promotion of GPX4 expression and the inhibition of iron uptake [107]. It has been demonstrated that DBP can exacerbate allergic asthma in ovalbumin (OVA)-sensitized mice. This is evidenced by the observation of increased airway hyperresponsiveness (AHR), more severe airway wall remodeling, and airway inflammation. These effects are attributed to the induction of lipid peroxidation and ferroptosis [110]. It is therefore anticipated that the targeted inhibition of ferroptosis will prove an effective means of mitigating the damage to the heart, liver, and respiratory system caused by exposure to phthalates.

#### Volatile organic pollutants (VOPs)

Volatile organic pollutants (VOPs) are air pollutants released from industrial activities, transportation, and building materials with significant impacts on human health. These include effects on the respiratory system, nervous system, reproductive health, and sex hormone levels [111, 112]. A comprehensive survey conducted in China (205 urine samples collected from 12 cities across the mainland) revealed that volatile organic compounds (VOCs), including benzene, isoprene, acrolein, and crotonaldehyde, were the primary contributors to oxidative stress in humans [113]. Excessive oxidative stress has been linked to the initiation of ferroptosis [114]. A study demonstrated that mice exhibited anemia of inflammation, characterized by decreased plasma Fe^2+^ and increased ferritin and inflammatory factors, following eight 8 weeks of benzene (50 ppm) exposure [115]. Moreover, benzene has been demonstrated to induce iron maldistribution in the spleen and bone marrow, which in turn promotes the expression of IRP1 and ALOX12, thereby eliciting inflammatory responses and ferroptosis [115]. Furthermore, another study has demonstrated that benzene treatment results in hematotoxicity, characterized by a reduction in the number of various blood cells, including leukocytes. Additionally, benzene has been observed to activate NRF2 and promote the expression of molecules downstream of NRF2, which in turn increases intracellular iron levels, lipid peroxidation, and redox imbalance, thereby inducing ferroptosis [116]. Formaldehyde is another VOP that induces neurotoxicity by promoting ferroptosis via nuclear receptor coactivator 4 (NCOA4)-mediated ferritinophagy in the HT22 mouse hippocampal neuronal cell line. This process is largely counteracted by hydrogen sulfide, which upregulates growth differentiation factor-11 (GDF11) expression [117]. It has been demonstrated that acrolein, a volatile organic compound (VOC) clearly associated with diabetes, can induce pancreatic β-cell ferroptosis by inducing endoplasmic reticulum (ER) stress and inhibiting peroxisome proliferator-activated receptor gamma (PPARγ) expression. It is noteworthy that resveratrol was observed to reverse the damage caused to pancreatic β-cells by acrolein [118]. Furthermore, 4-tert-butylphenol (4-tBP) exposure has been demonstrated to induce ferroptosis in hepatocytes by inhibiting the expression of SLC7A11, GPX4, ATF4, and HSPA5. This hepatotoxicity of 4-tBP has been shown to be reversed by Fer-1 [119]. These findings collectively suggest that VOPs may exert a detrimental impact on human health by promoting ferroptosis.

#### Others

It has been demonstrated that exposure to benzo(a)pyrene (BaP) or its metabolite BPDE (benzo(a)pyrene-7,8-dihydrodiol-9,10-epoxide) results in the expression of the E3 ligase MARCHF1, which in turn ubiquitinates and degrades GPX4. This process induces ferroptosis of human umbilical vein endothelial cells, disruption of angiogenesis, and miscarriage [120]. Furthermore, a number of environmental toxins have been demonstrated to exert an influence on human health through the modulation of ferroptosis. For example, rotenone is an insecticide that has been demonstrated to exhibit high levels of neurotoxicity and reproductive toxicity in mammals. Rotenone has been shown to downregulate the expression of SLC7A11, GPX4, and FTH1, thereby facilitating ferroptosis in mouse brain organoids [121]. Deoxynivalenol (DON) is a Fusarium toxin that promotes hepatocyte ferroptosis and causes liver injury by modulating the Nrf2/PPARγ-GPX4 pathway. In contrast, selenomethionine (SeMet) treatment has been demonstrated to significantly reverse DON-induced hepatotoxicity in mice [122]. Exposure to cyanobacterial toxins, such as microcystins (MCs), has been linked to the development of gastroenteritis and even gastric cancer through the regulation of ferroptosis [123].

In conclusion, it can be stated that organic pollutants have an adverse effect on human health. Ferroptosis is one of the major mechanisms through which organic pollutants induce damage to organs. Furthermore, the targeted inhibition of ferroptosis can effectively ameliorate the damage caused by various organic pollutants.

### Ferroptosis induced by inorganic pollutants and its health implications

In addition to organic pollutants, inorganic pollutants can also have a deleterious impact on human health, particularly in the context of heavy metal pollution. Cadmium (Cd) is a common heavy metal contaminant that has been demonstrated to cause organ damage through the activation of inflammatory processes, oxidative stress, mitochondrial damage, and cell death, which ultimately leads to neurotoxicity, gastrointestinal damage, renal damage, and reproductive damage [124, 125]. Cd exposure during puberty has been demonstrated to regulate the expression of FTH1, TFR1, and FPN1, leading to iron overload, ROS production, and GSH depletion in spermatogonia. This ultimately contributes to pathological testicular damage and sperm reduction in mice [126]. Moreover, both Fer-1 and DFO were demonstrated to be effective in reversing Cd-induced damage to the male reproductive system [126]. Chronic Cd exposure has been demonstrated to induce renal damage by activating ferroptosis [127]. A multiomics analysis revealed that Cd induces lysosomal iron overload by upregulating STEAP3 expression, which results in decreased glutathione and increased lipid peroxidation in mouse kidneys [127]. Furthermore, exposure to Cd has been demonstrated to cause injury to the cerebrum and cerebellum in swine by regulating ferroptosis and necrosis [124]. The small particle size, high hydrophobicity, and extensive distribution of MPs render them capable of adsorbing or desorbing a diverse range of environmental contaminants, including Cd [128, 129]. The concurrent exposure to PS-MPs and Cd has been observed to downregulate the expression of miR-199a-5p, which in turn has been demonstrated to increase the expression level of HIF-1α, thereby promoting ferroptosis and impairing male reproductive function [129]. Similarly, Lan et al. demonstrated that co-exposure to PS-MPs and Cd activates ferroptosis, thereby causing male reproductive toxicity via the suppression of the Keap1-Nrf2 pathway in mice [130]. Moreover, concomitant exposure to Cd and PS-NPs has been demonstrated to induce renal damage through the inhibition of the Nrf2 pathway and the activation of excessive mitophagy [125]. These findings indicate that Cd, in addition to exerting adverse effects on human health when present alone, can also act in a synergistic manner with MPs to enhance the overall detrimental impact on the body.

In addition to cadmium, antimony, arsenic, and nickel have been demonstrated to affect health through modulation of ferroptosis. For example, exposure to antimony (Sb) has been observed to promote the expression levels of heat shock cognate protein 70 (HSC70), heat shock protein 90 (HSP90), and lysosomal-associated protein 2 A (LAMP2A). These proteins bind to GPX4 and transport it to lysosomes in a chaperone-mediated autophagy manner, resulting in GPX4 degradation and ferroptosis. This ultimately leads to neurotoxicity [131]. In contrast, Shi et al. demonstrated that low-dose antimony exposure facilitates the expression of Nrf2, which then upregulates its targets SLC7A11 and GPX4, thereby inhibiting ferroptosis and accelerating prostate cancer progression [132]. Additionally, the researchers discovered that prostate cancer patients exhibited elevated serum antimony levels in comparison to individuals with benign prostatic hyperplasia. Furthermore, they observed that elevated serum antimony concentrations were associated with a poorer prognosis [132]. Arsenic exposure has been demonstrated to activate Nrf2 expression while simultaneously inhibiting GPX4 expression, thereby inducing ROS accumulation and ferroptosis. This ultimately results in liver injury in rats [133]. The administration of *Rosa roxburghii Tratt* (RRT) was observed to partially reverse the damage caused to the liver by arsenic exposure [133]. On the other hand, arsenic exposure has been demonstrated to activate the transcriptional activity of HIF-2α, which in turn promotes dual oxidase 1 (DUOX1) expression. This subsequently inhibits the expression of GPX4, thereby inducing ferroptosis and resulting in kidney injury [134]. Salvianolic acid A has been demonstrated to mitigate the adverse effects of arsenic exposure on the kidney [134]. Furthermore, nickel exposure has been demonstrated to induce developmental neurotoxicity by regulating ferroptosis in zebrafish [135].

In conclusion, inorganic pollutants exert a considerable influence on human health, particularly through the modulation of ferroptosis and the consequent damage to the liver and kidneys. Furthermore, inorganic pollutants (such as cadmium) may act in a synergistic manner with organic pollutants (such as microplastics) to amplify the adverse effects on the body. It is anticipated that the targeted inhibition of ferroptosis will serve to mitigate the deleterious effects of inorganic and organic pollutants.

### Ferroptosis induced by ionizing radiation and its health implications

Ionizing radiation is a pervasive tool in the fields of medical imaging (e.g., radiography, nuclear medicine, and computed tomography (CT)) and in radiation therapy for tumors. Its utilization has precipitated significant advancements in the diagnosis and treatment of human diseases [136]. To illustrate, radiotherapy results in mitochondrial damage and mitophagy, which releases free fatty acids and increases lipid peroxidation, thereby inducing ferroptosis in tumor cells [137]. The knockdown of ribonucleotide reductase subunit M1 (RRM1) has been observed to inhibit the expression of the deubiquitinating enzyme USP11 while simultaneously promoting the expression of the ubiquitinating enzyme MDM2. This results in an enhanced interaction between MDM2 and p53, as well as increased ubiquitination of p53 in tumor cells. The degradation of p53 leads to a reduction in p21 expression, which in turn inhibits the expression of GPX4 and promotes lipid peroxidation and ferroptosis, thereby increasing the sensitivity of radio/chemotherapy [138]. However, the study did not yield evidence that p53 or p21 interacts with GPX4 proteins in HCT116 and HeLa cells, suggesting that p53 or p21 do not directly regulate GPX4 stability [138]. Conversely, Zhang et al. demonstrated that P21 influences the stability of the GPX4 protein by modulating its recruitment to the linear ubiquitin chain assembly complex (LUBAC), thereby regulating the level of M1-linked ubiquitination of GPX4 in the pathogenesis of osteoarthritis [139]. The discrepancy may be attributed to differences in stimuli and cells. Although ionizing radiation employed for medical purposes is typically low-dose, long-term medical radiation exposure or high-dose exposure has been linked to adverse effects on human health, including digestive system diseases and circulatory and metabolic diseases [140, 141]. It has been demonstrated that programmed cell death, particularly ferroptosis, is associated with health issues linked to ionizing radiation.

#### Digestive systems

Radiation-induced intestinal injury (RIII) is a prevalent gastrointestinal complication resulting from radiation therapy for cancer patients, particularly for pelvic, abdominal, and retroperitoneal tumors. It is frequently accompanied by diarrhea, abdominal discomfort, nausea, and vomiting. [142]. RIII has a markedly deleterious impact on the quality of life of patients undergoing radiation therapy, potentially resulting in the cessation of radiation therapy due to intolerance [143]. Irradiated mice exhibited a notable reduction in intestinal villus height and crypt depth, accompanied by an activated inflammatory response, as evidenced by elevated expression of IL-6, TGF-β1, TNF-α, and IFN-γ [144, 145]. Furthermore, radiation exposure was observed to elevate iron and MDA levels, upregulate LPCAT3 and ALOX15 expression, and disrupt or eliminate cristae in small intestinal mitochondria when compared to the control group. These findings suggest that ferroptosis may play a role in the pathogenesis of radiation-induced intestinal injury [145]. Mechanistically, radiation treatment has been observed to promote STAT1 phosphorylation, which in turn activates the STAT1-IRF1 axis and facilitates the expression of ACSL4. This ultimately renders cells more susceptible to lipid peroxidation and ferroptosis [143]. A report indicated that Pseudomonas aeruginosa (PAO1), a Gram-negative bacterium, exacerbates radiation-induced ferroptosis by utilizing bacterial 15-lipoxygenase to catalyze the production of 15-hydroperoxy-arachidonoyl-PE (15-HpETEPE). This effect was reversed by the lipoxygenase inhibitor baicalein [146]. It is of particular importance to note that the administration of total flavonoids derived from Engelhardia roxburghiana Wall. leaves (TFERL) have been observed to mitigate radiation-induced ferroptosis and intestinal structural damage in mice via the activation of the NRF2 pathway [142]. It is therefore anticipated that the inhibition of ferroptosis in intestinal cells will prove an effective means of mitigating the side effects of radiotherapy in patients.

#### Circulatory system

A high dose of total body irradiation results in an acute hematopoietic injury, characterized by a reduction in peripheral haematocytes, including red and white blood cells, monocytes, and lymphocytes [147]. It has been demonstrated that ferroptosis plays a role in the loss of peripheral haematocytes following total body irradiation, with increased iron and lipid peroxidation levels observed in mice [147]. Radiation therapy has been demonstrated to exert adverse effects on the cardiovascular system, thereby exacerbating the progression of cardiovascular disease, including atherosclerosis [148, 149]. Ionizing radiation has been demonstrated to activate ferroptosis of endothelial cells by enhancing P38/NCOA4-dependent ferritinophagy, thereby accelerating the pathological progression of atherosclerotic plaques [148]. Furthermore, thoracic radiotherapy frequently results in lung injury, which can be mitigated by the ferroptosis inhibitor liproxstatin-1 in murine models [150]. The P62-Keap1-NRF2 pathway has been demonstrated to exert a protective influence with respect to radiation-induced ferroptosis and lung injury [151]. The bioengineered nanoreactor SOD@ARA290-HBc has been demonstrated to offer protection against radiation-induced pneumonitis and lung fibrosis by virtue of its capacity to inhibit ferroptosis and apoptosis [152].

In conclusion, there is evidence to suggest that ferroptosis plays a role in the damage caused by ionizing radiation, particularly radiotherapy, to the digestive and circulatory systems. The targeted inhibition of ferroptosis is anticipated to attenuate the damage caused by ionizing radiation. However, it is paradoxical that radiotherapy also results in the death of tumor cells by activating ferroptosis. It is therefore evident that the key to reducing the side effects of radiotherapy on other tissues and increasing the tumor-killing effect lies in the development of targeted therapy.

## Conclusions and perspectives

The presence of environmental pollutants, including atmospheric pollutants such as PM2.5, organic compounds such as MPs and bisphenol A, inorganic substances such as heavy metals like cadmium and arsenic, and ionizing radiation, has been linked to a wide range of adverse health effects. These effects encompass abnormalities in heart development, reproductive toxicity, lung injuries, neurotoxicity, hepatotoxicity, and carcinogenesis. The mechanisms by which these pollutants exert their toxic effects are multifaceted, involving a complex interplay of molecular pathways that compromise cellular integrity and organ function. Among these mechanisms, recent studies have highlighted ferroptosis as a critical pathway in the pathogenesis of diseases induced by environmental pollutants. Ferroptosis, a unique form of programmed cell death, is driven by the dysregulation of iron metabolism, antioxidant systems, lipid metabolism, and lipid peroxidation. The major pathways that regulate ferroptosis include the xCT-GPX4-GSH, FSP1-CoQ, DHODH-CoQ, and 7-DHC pathways. It has been demonstrated that environmental pollutants induce ferroptosis by disrupting iron homeostasis, antioxidant defenses, and lipid metabolism. Recent advances in ferroptosis research offer promising avenues for therapeutic intervention. Studies indicate that targeting ferroptosis-related pathways could partially reverse the damage caused by a variety of pollutants, including PM2.5, organic pollutants, and ionizing radiation. For instance, ferroptosis inhibitors, such as iron chelators, ferrostatin-1, and liproxstatin-1, have shown potential in experimental models to mitigate pollutant-induced tissue injury. By restoring iron homeostasis, enhancing antioxidant defenses, and preventing lipid peroxidation, these agents may offer protective effects against the health impacts of pollutants. In conclusion, ferroptosis is emerging as a pivotal mechanism underlying the toxic effects of environmental pollutants on human health. Understanding how specific pollutants trigger ferroptosis provides critical insights into the pathogenesis of pollution-related diseases and highlights potential therapeutic targets. As research progresses, there is hope that interventions targeting ferroptosis could serve as effective strategies to mitigate the adverse health effects of environmental pollutants and improve outcomes for affected populations.

Despite recent evidence indicating that ferroptosis plays a significant role in the adverse effects of environmental pollutants on organisms, several key questions remain unanswered, including: (1) It would be beneficial to ascertain which environmental pollutants cause damage to the organism that depends on ferroptosis. (2) What is the mechanism by which environmental pollutants induce ferroptosis? (3) It would be beneficial to ascertain whether there is a specific molecular mechanism underlying the induction of ferroptosis by environmental toxicants. (4) It would be of interest to ascertain whether novel molecular mechanisms regulating ferroptosis are involved in the damage to organs caused by environmental contaminants. (5) Which environmental pollutants cause damage to the organism that could be prevented by targeting ferroptosis? (6) In the context of radiotherapy for cancer patients, how might ferroptosis be regulated to mitigate the adverse effects of radiotherapy without compromising its tumor-killing efficacy? (7) The majority of current research focuses on single environmental pollutants, despite the fact that damage to organisms is often caused by multiple pollutants. This raises the question of whether targeted ferroptosis is still an effective method of mitigating the damage caused by the interactions between pollutants. Ferroptosis is currently understood to be one of the most important mechanisms by which environmental pollutants damage organisms. However, despite the lack of resolution regarding the effectiveness of ferroptosis as a method of mitigating damage caused by pollutants, the discovery of this mechanism provides new avenues for intervention in the effects of pollutants on organisms.
